# Do the Sick Fear Death?

**Published:** 1890-06-07

**Authors:** 


### Do the Sick Fear Death?

By a Consulting Physician.

"Do the sick fear death?" is one of those questions that must have crossed the mind of many a medical man, if not of every one, and is, moreover, one of so probing and heart-searching a nature that I think there can be few of us who have not thought over it and answered for himself. It is a question that has long interested me very deeply, and I read the article in a late number of The Hospital with avidity. I gather that the writer is not a medical man [He is.—Ed.], for I cannot think that any of that calling could speak in any other way than the few have already done, as the article in question tells us. For myself I answer the question unhesitatingly in the negative. Many years ago, when resident medical officer of one of our largest metropolitan hospitals, I made it my practice to be called to every patient as he or she appeared to the nurses to be nearing death, and ever since then, now two-and-twenty years ago, when the sad opportunity has been afforded me, I have watched the process of dying in more cases than I like to think of. The result is a conviction that admits of no doubt, and not only so, it leaves me no room for thinking that any one who has given any heed to the matter can possibly come to any different conclusion tomyown. The healthy fear death; the sick of trivial ailments oftentimes still more so; but those who are dying have no fear of death in the vast majority of cases, because the question, "Am I going to die?" seldom comes instantly and persistently before them. There is a terrible phrase among the common people, "he died hard." I once saw a man die hard; it was some ten years ago, and I shall never forget it, but thank God I have not seen another. That poor man knew he was dying; realised the fact; and the future was a blank. But cases of that sort are the rarest exceptions. In the large proportion that are not accidents or sudden, dying is a process not an act. We may be taken with acute disease in the midst of seeming health: in such case death has no terror, because we know not of its on-coming. In such, perhaps, it may be said, as the writer of your article puts it, that the struggle to live obscures the approaching end. But in the many cases of chronic disease the cessation of life is a gradual one, and day by day, as the vital energies ebb, the mental power of realising in its full intensity the magnitude of the issue becomes dulled in similar degree. This must be so. As long as life remains, we are subject to physiological law; and it is very exceptional that serious disease leaves its subject with acuteness of mind sufficient to realize that death is imminent. There is often the knowledge of serious illness, but to this is added the feeling of weariness, of drowsiness, of mental torpor, and thus the Present with its longing for rest, and the cessation or mitigation of distressing symptoms, and if they are happily mitigated the pleasure of the ease, obscure the Future.

But it will be said that there are many cases where the mental faculties are retained to the last. For the sake of argument I shall admit this to the full, although I do not think that there are many who retain their mental acuteness at the healthy standard, as the phrase would imply. But there are many who, in lingering illness—consumption, for example—are conscious of their earthly habitation, and have full knowledge of their friends, to the last. These, however, have no fear of death, because, as with the others, they too fail to realize that death is at hand. They have often a knowledge that a mortal disease affects them, and that they will die some time, but for the present they are hopeful of living, and death never comes to them as a certainty of the immediate future, and there is no terror as long as there is hope. No! I look around and into the vista of sickness unto death, and I see little real fear of the King of Terrors depicted in the faces of the dying; and the "agony of death" is one for those who are watching, not for him that is leaving that night or that day on his strange, strange journey. And this is not because the traveller steps out with the firm footstep of one who knows whither he is going, as some will think and say; it is not because he knows, in the beautiful language of religious consolation, that he is going home—would that one could say so, but it is beside that question, for I am speaking only of the physical side of death—but it is because, in the merciful providence of God, we cannot die otherwise than as we live, and the varied conditions that consummate death blunt our perceptive faculties, in common with those other functions which constitute us living beings, and that then as ever it is still:—"the day and the hour knoweth no man." This is so, and must be so, except in exceptional cases.

But I have not written this merely from the interest of the subject, but because it is closely related to another question which perplexes, and rightly so, us doctors. It is this: Ought we to tell a patient that he is going to die when the prospect seems to us hopeless? The answer to that question involves a tremendous issue, and a responsibility than which few can be greater. If I ask it of the non-professional person, and away from real life, so to speak, as an interesting subject for discussion, I shall probably be answered without hesitation in the affirmative. If at the bedside, or rather under the shadows of past or present illness, the answer is less positive. I then hear, "Oh! yes, he must be told," or, "It was cruel of the doctor not to tell him"; or again, "Don't tell him; he is so nervous about himself," which is in itself evidence, if it be true, that the sick do fear death; but I have already anticipated that objection. How ought we to act under such circumstances? Is it the right thing to try to make a sick man realize that he is going to die when of his own instance he does not realize it? The answer to this question must, in some degree, depend upon the patient, for no doubt there are some who know their own minds, so to speak, even at that anxious time. Nevertheless, I think that such are quite the exception, and in my experience the sick in great measure divide into those who cannot be made to realize their critical position, either because they are too ill, or because their lives are too instinct with hope, when in either case to tell them is of no avail; and those who when they realize the whole truth are depressed to such a degree, as from a physical point of view to be made the worse for it. Add to this that there are many even desperately ill who cannot be said by the physician to be beyond a ray of hope, and the conclusion I have arrived at is that the sick man himself is seldom the right one to whom to convey an irrevocable verdict of this kind.

